# Molecular Iodine Induces Anti- and Pro-Neoplastic Effects in Prostate Cancer Models

**DOI:** 10.3390/ijms26167800

**Published:** 2025-08-13

**Authors:** Carlos Montes de Oca, Lourdes Álvarez, Carmen Aceves, Brenda Anguiano

**Affiliations:** Departamento de Neurobiología Celular y Molecular, Instituto de Neurobiología, Universidad Nacional Autónoma de México, Campus Juriquilla, Querétaro 76230, Mexico; carlos.f.montesdeoca@gmail.com (C.M.d.O.); malualvarez423@gmail.com (L.Á.); caracev@unam.mx (C.A.)

**Keywords:** prostate, iodine, PPARG, TRAMP, androgen deprivation, lipids

## Abstract

Advanced prostate cancer frequently develops resistance to antiandrogen therapy and acquires an aggressive neuroendocrine phenotype. Antiandrogens stimulate peroxisome proliferator-activated receptor gamma (PPARG) signaling and cancer progression. Molecular iodine (I_2_) induces cytotoxic effects in prostate cancer cell lines and antineoplastic effects in neuroblastoma and breast cancer through the indirect activation of PPARG. We investigated the adjuvant effects of I_2_ and androgen deprivation in prostate cancer, as well as the role of PPARG in these projections. We used androgen-dependent and androgen-independent cell lines and TRAMP mice (transgenic adenocarcinoma of the mouse prostate) as biological models, as well as bicalutamide (Bic), enzalutamide (Enz), and charcoal-stripped fetal bovine serum (CS-FBS) as androgen deprivation models. I_2_ promoted cytotoxic effects, whereas in surviving cells, it stimulated the outgrowth of neurite-like projections, regulated lipid content, and reduced invasive capacity. Androgen deprivation plus I_2_ magnified these effects, while GW9662 (PPARG antagonist) did not block them. In vivo, I_2_ increased the degree of prostatic desmoplasia in the sham mice but did not amplify the stromal response or reduce the epithelial lesion score induced by castration in TRAMP. In conclusion, I_2_ showed anti-cancer (cytotoxic, anti-invasive) and pro-cancer (pro-neurite, lipid accumulation, desmoplasia) effects through a PPARG-independent mechanism.

## 1. Introduction

Prostate cancer is the second most diagnosed neoplasia and fifth leading cause of cancer-related death among men worldwide [1]. Advanced prostate cancer is generally treated with the pharmacological inhibition of androgen receptor (AR) signaling [2]. However, cancer cells develop resistance mechanisms in about 25% of cases, and tumors progress to a lethal metastatic form [3]. The neuroendocrine (NE) transdifferentiation of prostate adenocarcinoma is part of the cellular plasticity mechanisms that occur in response to androgen deprivation therapy, and leads to gradual changes characterized by the loss of epithelial identity (loss of AR/downstream genes) and the acquisition of NE features (neuropeptide production) [4,5]. NE-like prostate cancer is highly metastatic and responds poorly to therapies [6]. Hence, a better understanding of the mechanisms of tumor progression will allow us to identify possible therapeutic targets and improve combination strategies.

Iodine is an essential micronutrient for thyroid hormone synthesis and extra-thyroid actions [7]. The administration or consumption of molecular iodine (I_2_) has been shown to induce antineoplastic effects on breast, neuroblastoma, thyroid, and cervical cancer models by promoting apoptosis and reducing cell migration and epithelial–mesenchymal transition [8,9,10]. The mechanisms of I_2_ involve direct antioxidant action and the formation of an iodinated lipid (6-iodolactone) that functions as a ligand for peroxisome proliferator-activated receptor gamma (PPARG) [11]. Prostate and prostate cancer cells capture I_2_, which exerts antioxidant, anti-inflammatory, and apoptotic actions depending on the cellular context [12,13,14]. Although 6-iodolactone induces apoptosis in prostate cancer cell lines [14], the anti-proliferative actions of I_2_ do not appear to be mediated by PPARG in androgen-independent cells [15]. PPARG possesses two transcriptionally active forms with opposite effects [16]. PPARG1 enhances cell proliferation, lipid synthesis, and metastasis, correlating with low survival [17]. In contrast, PPARG2 reduces cell proliferation and migration and triggers an anti-tumor inflammatory response in prostate cancer models [18]. Moreover, a complex crosstalk between the AR and PPARG has been identified, in which PPARG1 activation reduces AR transcriptional activity, whereas PPARG2 facilitates AR signaling [19,20,21]. On the other hand, in advanced prostate cancer, the androgen receptor blockade enhances PPARG lipogenic signaling and the acquisition of a prostate NE phenotype [22], revealing mutual interrelationships.

This study aimed to analyze the individual or combined effects of I_2_ and androgen deprivation on prostate cancer and to assess the role of PPARG in these projections. We hypothesized that I_2_ enhances the anticancer effects of androgen antagonists under conditions of androgen deficiency. To test this, we evaluated cell viability, invasive capacity, and the acquisition of a NE-like phenotype in prostate cancer cell lines cultured under androgen-deprived conditions and in the presence of a PPARG selective antagonist. We also analyzed the expression of key genes involved in AR signaling (*KLK3*, prostate-specific antigen; *NKX3-1*, NK3 homeobox 1) and PPARG-regulated lipid metabolism (*FASN*, fatty acid synthase; *SREBF1*, sterol regulatory element binding transcription factor 1), as well as neuroendocrine differentiation markers (*SYP*, synaptophysin; *ENO2,* enolase 2). In the in vivo TRAMP (transgenic adenocarcinoma of the mouse prostate) model, we analyzed the histopathological changes and immunolocalization of AR, PPARG, and SYP in response to I_2_ and/or androgen deprivation.

## 2. Results

### 2.1. I_2_ Decreases Cell Viability and Invasive Capacity in Prostate Cancer Cell Lines

**Figure 1:**
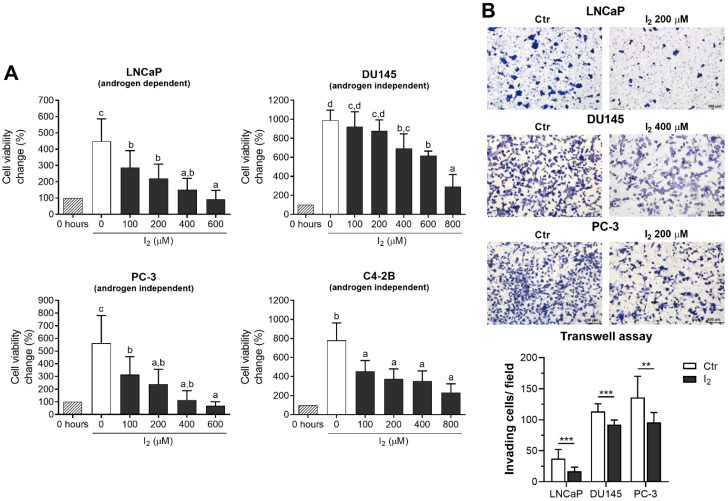
The effects of I_2_ on prostate cancer cell proliferation and invasive capacity. (**A**) LNCaP, DU145, PC-3, and C4-2B cells were cultured in increasing I_2_ concentrations. Cell viability was assessed at 96 h of treatments and was normalized against the initial cell population (0 h). (**B**) Representative micrographs and quantification of the invasion assay on prostate cancer cells pretreated with I_2_ for 48 h. Cell numbers were adjusted for the Transwell assays. Invading cells were dyed with toluidine blue and counted six hours later at 100× magnification. All data represent mean ± SD, *n* = 4–6 independent experiments in duplicate. Cell viability was analyzed with one-way ANOVA followed by Tukey post hoc tests. Statistical differences among groups are indicated with different letters (*p* < 0.05); groups that share the same letter are not significantly different. Transwell assays were analyzed with an unpaired *t*-test (** *p* < 0.01, *** *p* < 0.001).

We analyzed the effects of I_2_ in both androgen-dependent cells (LNCaP) and androgen-independent cells (DU145, PC-3, and C4-2B). I_2_ decreased cell viability in a dose-dependent manner across the four cell lines. In LNCaP, PC-3, and C4-2B cells, a reduction of ~50% was observed at 200 µM I_2_; in contrast, DU145 cells only exhibited a ~20% reduction at that concentration (Figure 1A). Based on this lower sensitivity, we used 400 µM I_2_ for the subsequent functional assays involving DU145 cells. Consistently, I_2_ reduced the invasive capacity of cell lines with low (LNCaP) or high (DU145, PC-3) invasive potential by 55% and ~30%, respectively (Figure 1B).

### 2.2. The Effects of I_2_ and Androgen Antagonists on the Loss of Cell Viability in LNCaP Cells

**Figure 2:**
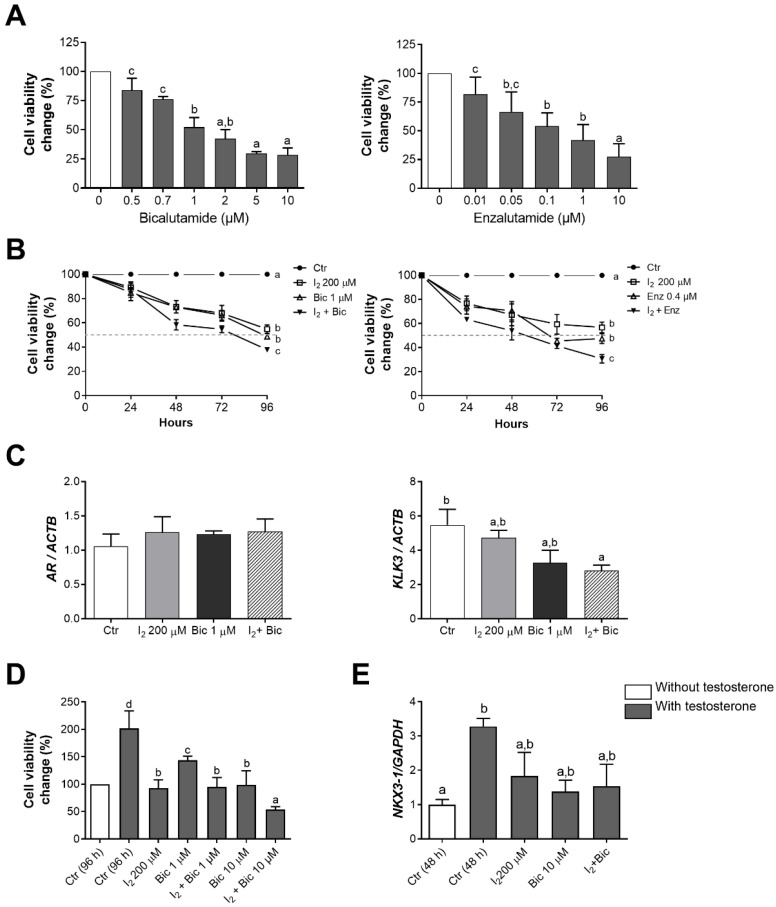
The effects of I_2_ and antiandrogens on LNCaP cells cultured in standard or androgen-stimulated conditions. (**A**) Dose-response effect of the androgen receptor antagonists bicalutamide (Bic) or enzalutamide (Enz) on cell viability at 96 h. (**B**) Time course and adjuvancy of the individual and combined effects of I_2_ and antiandrogens. (**A**,**B**) Cells were cultured for 96 h in standard 10% FBS supplemented medium, with I_2_ and/or Bic or Enz. Cell viability was normalized against its respective control. (**C**) Real-time PCR analysis of the androgen receptor (*AR*) and prostate-specific antigen (*KLK3*) expression in cells treated for 96 h. (**D**) Cell viability assays evidenced an adjuvancy of I_2_ and Bic in cells cultured with 10 nM of testosterone for 96 h. (**E**) The expression of androgen-responsive gene (*NKX3-1*) in LNCaP-treated cells for 48 h under androgen-stimulated conditions. *n* = 3–6 independent experiments in duplicate. All data represent mean ± SD. Statistical differences among groups are indicated with different letters (*p* < 0.05); groups that share the same letter are not significantly different.

Our results demonstrated the capacity of AR antagonists bicalutamide (Bic) and enzalutamide (Enz) to decrease cell viability in a dose-response manner (Figure 2A). We found that individual treatments of 200 µM I_2_, 1 µM Bic, or 0.4 µM Enz reduced cell viability by around 50%, whereas AR antagonists combined with I_2_ exerted enhanced effects (Figure 2B). However, when analyzing the specific effects of Bic or I_2_ over the AR signaling pathway after 96 h of treatment, no changes were observed in the AR expression under any condition. I_2_ + Bic significantly reduced *KLK3* expression in comparison to the control group (Figure 2C).

Androgens regulate cell proliferation and differentiation of the prostate epithelium [23]. In this study, we analyzed how I_2_ and/or Bic influence cell proliferation and the expression of an AR-regulated epithelial differentiation gene (*NKX3-1*) under androgen stimulation.

We found that testosterone (10 nM) doubled the cell population at 96 h of treatment in comparison to cells lacking androgen. This increment was prevented by the co-administration of 200 µM I_2_ and either 1 or 10 µM Bic. A reduction below the initial cell population was seen in the I_2_ + 10 µM Bic group (Figure 2D). In contrast, neither I_2_ nor Bic significantly prevented the increased expression of *NKX3-1* in testosterone-stimulated LNCaP cells (Figure 2E).

### 2.3. I_2_ Enhanced the Outgrowth of Neurite-like Projections Induced by Androgen Deficiency

**Figure 3:**
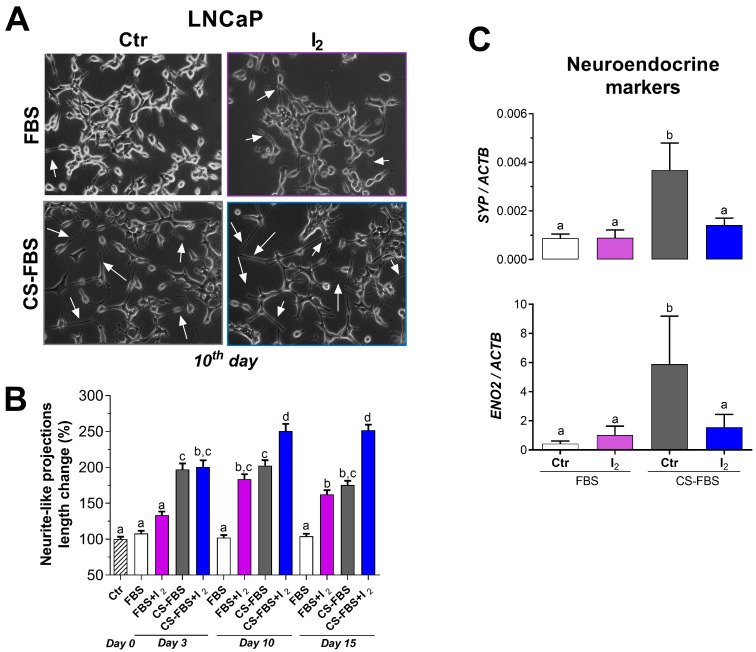
The effects of I_2_ and androgen deprivation on the growth of neurite-like projections and neuroendocrine marker gene expression. (**A**) Representative micrograph of the cells supplemented for 10 days with 10% FBS or 10% CS-FBS (androgen deprivation). Neurite-like projections are depicted (arrows). Images were captured at 20× magnification using a bright-field microscope. (**B**) The time course of neurite outgrowth. The length of projections was normalized against the basal length at initial time (0 h). Data represent mean ± SE. *n* = 3 independent experiments. Data were analyzed with a two-way ANOVA followed by a Tukey post hoc test. (**C**) Real-time PCR expression of the neuroendocrine markers *SYP* and *ENO2* in LNCaP cells on the 10th day of treatments. *n* = 4 independent experiments in duplicate and normalized by beta-actin (*ACTB*). Data represent mean ± SD and were analyzed with a one-way ANOVA followed by a Tukey post hoc test. Statistical differences among groups are indicated with different letters (*p* < 0.05); groups that share the same letter are not significantly different.

As expected, androgen depletion induced by 10% charcoal-stripped fetal bovine serum (CS-FBS) supplementation reduced the viability (~5-fold) of LNCaP cells cultured for 96 h and induced the growth of neurite-like projections in cultures measured at 12, 17, and 21 days (Supplementary Figure S1A–C). The time course analysis indicated that both androgen deprivation and I_2_ supplementation individually induced the growth of neurite-like projections on days 3 and 10, respectively, with an enhanced effect observed on days 10 and 15 (Figure 3A,B). In contrast, I_2_ alone did not alter the expression of *SYP* or *ENO2* but inhibited the expression of NE markers elicited by CS-FBS supplementation in cells cultured for 10 days (Figure 3C).

### 2.4. I_2_ Effects Are Not Mediated by PPARG in the LNCaP Cell Line

**Figure 4:**
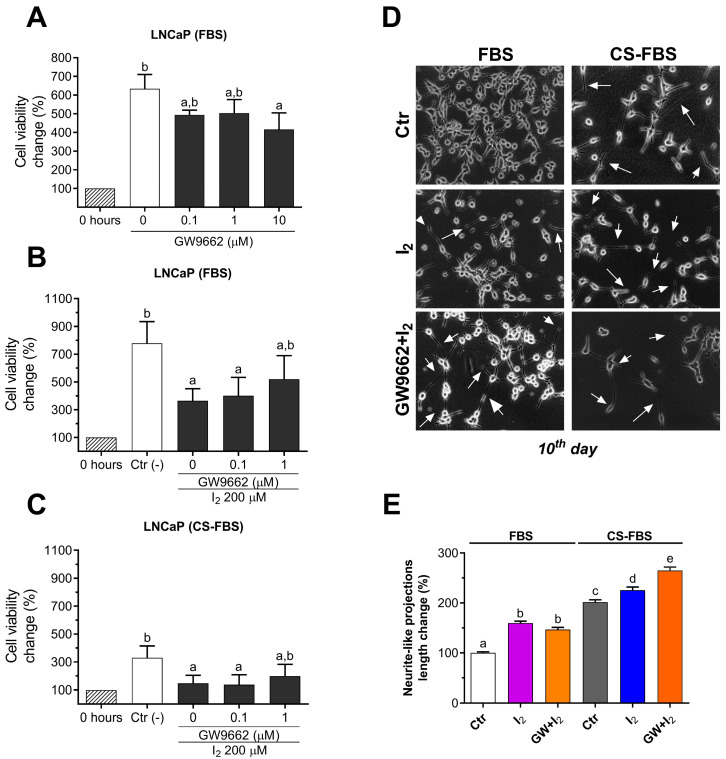
The participation of PPARG in I_2_ anti-proliferative effects and the growth of neurite-like projections. (**A**) Dose-response of the PPARG antagonist (GW9662) in LNCaP cells supplemented with fetal bovine serum (FBS) and treated for 96 h. (**B**,**C**) LNCaP cells supplemented either FBS or charcoal-stripped fetal bovine serum (CS-FBS) were treated with I_2_ and increasing doses of GW9662 for 96 h. Cell viability was normalized against the non-treated control group at 0 h. Data represent mean ± SD and were analyzed with a one-way ANOVA followed by a Tukey post hoc test; *n* = 3–4 independent experiments. (**D**) Representative micrographs of LNCaP cells treated for 10 days, highlighting the growth of neurite-like projections (arrows); 1 µM GW9662 was applied as indicated. Images were captured at 20× magnification using a bright-field microscope. (**E**) Comparison of the neurite-like projections in 10-day cultures. GW9662 is abbreviated as GW. Data represent mean ± SE and were analyzed with a one-way ANOVA followed by a Tukey post hoc test. *n* = 3 independent experiments. Different letters indicate statistical differences among groups (*p* < 0.05).

Given that numerous studies have attributed PPARG activation as the main effector of I_2_ actions, we assessed the role of PPARG on I_2_ effects regarding cell viability and neurite-like projection outgrowth. To this end, we co-cultured LNCaP cells with a selective PPARG antagonist (GW9662). The dose-response analysis showed that concentrations between 0.1–1 µM GW9662 reduced the viable cell population by ~20%, whereas 10 µM GW9662 reduced it by ~30% (Figure 4A). Next, when we simultaneously administered I_2_ plus increasing doses of GW9662, we found that concentrations between 0.1 and 1 µM GW9662 did not block the anti-proliferative effects of I_2_ (Figure 4B,C) in FBS- or CS-FBS–supplemented LNCaP cells, nor the growth of neurite-like projections (Figure 4D). Moreover, the combination of GW9662 + I_2_ enhanced the pro-neurite effect compared to I_2_ alone (Figure 4E).

### 2.5. The Lipidic Response by Iodine Is Not Mediated by PPARG

**Figure 5:**
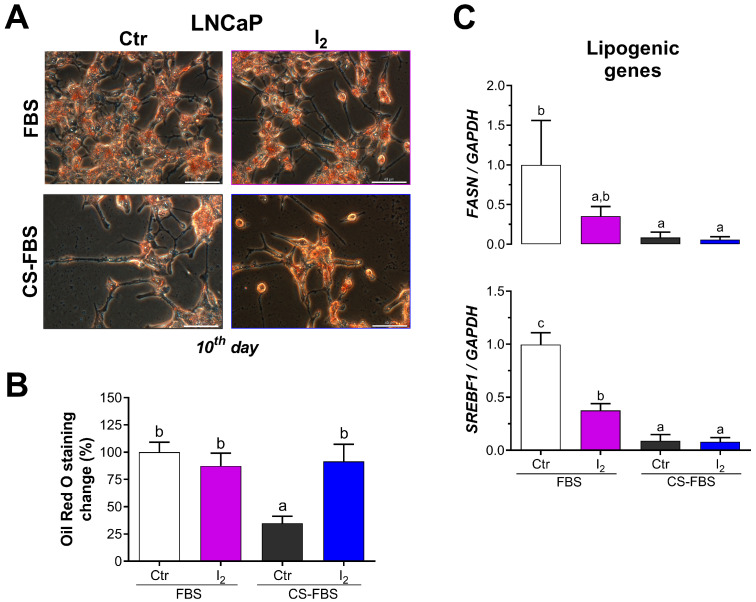
Lipogenic pathway analysis of iodine-treated LNCaP cells. Cells were cultured for ten days in standard or androgen depleted conditions with or without I_2_. Afterwards, cells were fixed and their intracellular neutral lipids analyzed with Oil Red O staining. (**A**) Representative micrograph of the stained cells. Notice that the lipids are primarily localized within the cell body and not in neurite-like projections. Scale bar: 40 μm. (**B**) Densitometric quantification of stained neutral lipids; *n* = 3, independent experiments. (**C**) Real-time PCR expression of the PPARG target genes fatty acid synthase (*FASN*) and sterol regulatory binding transcription factor (*SREBF1*) in LNCaP cells treated with 200 µM I_2_ for 48 h. All data represent mean ± SD and were analyzed with a one-way ANOVA followed by a Tukey post hoc test; *n* = 3–4 independent experiments. Statistical differences among groups are indicated with different letters (*p* < 0.05); groups that share the same letter are not significantly different.

As PPARG signaling is widely known to mediate adipose metabolism through the transcription of lipogenic enzymes, we explored whether I_2_ could modulate the lipid content in surviving NE-like LNCaP cells. We found that androgen deprivation decreased the content of neutral lipids, whereas I_2_ supplementation had no effect under standard conditions; nonetheless, it prevented lipid decline due to androgen deprivation (Figure 5A,B). Consistently, I_2_ also increased the lipid content in androgen-independent DU145 cells (Supplementary Figure S2). Paradoxically, I_2_ reduced or showed a tendency to reduce the expression of *FASN* and *SREBF1,* respectively, which are PPARG-responsive lipogenic genes (Figure 5C).

### 2.6. Effects of I_2_ and/or Castration on Histology and Prostate Pathology

**Figure 6:**
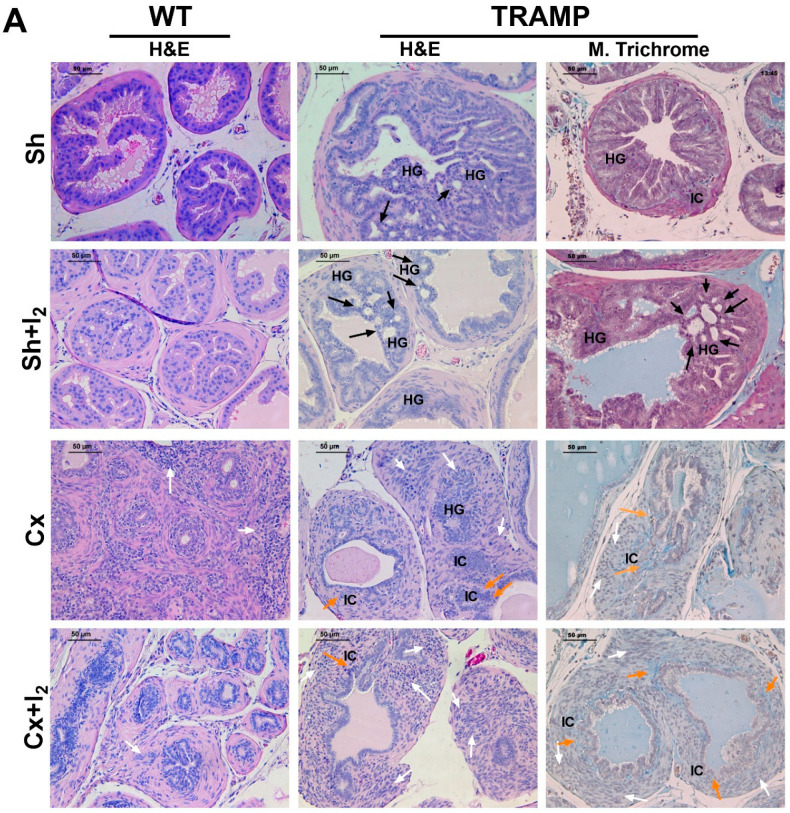

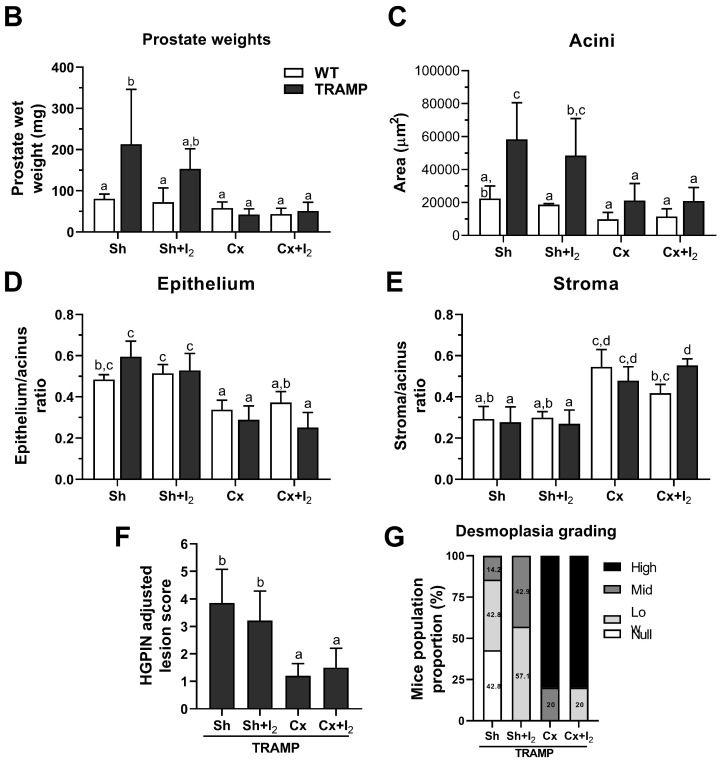
Analysis of I_2_ and/or castration effects on normal and cancerous prostates. (**A**) Representative micrographs of normal (WT) and cancerous (TRAMP) prostates. Tissues were stained through hematoxylin–eosin (H&E) and/or Masson’s trichrome. Black arrows indicate high-grade prostatic intraepithelial neoplasia (HGPIN) with cribriform features. White arrows highlight stromal hyperplasia (desmoplasia). Orange arrows indicate basal membrane integrity loss. HG: high-grade prostatic intraepithelial neoplasia; IC: invasive carcinoma. All images were captured at 20× magnification under light microscopy; scale bar equals 50 µm. (**B**) Comparison of prostate wet weights among groups. (**C**–**E**) Prostate histological parameters: acinus area, epithelium/acinus and stroma/acinus ratios. (**F**,**G**) Prostate pathological analysis: HGPIN epithelial lesion scores and desmoplasia prevalence in TRAMP mice. Data represent SD and were analyzed with one-way or two-way ANOVA followed by Tukey post hoc tests. *n* = 5–7 mice/group. Four to five independent fields per mouse were analyzed. Statistical differences among groups are indicated with different letters (*p* < 0.05); groups that share the same letter are not significantly different. Sham (Sh), Castration (Cx).

This experiment was performed to determine if the anti-proliferative and anti-invasive effects of I_2_ on prostate cancer cells could improve the antitumor effects of androgen deficiency in vivo. For this purpose, TRAMP mice were deprived of androgens by surgical castration (Cx) and subsequently supplied with I_2_ in their drinking water (0.025%). Representative micrographs depict prostate acini in wild-type (WT) and TRAMP mice, which were subdivided into Sham (Sh) and Cx groups (Figure 6A). As expected, the Sham TRAMP (Sh-TRAMP) mouse prostate acini displayed hyperchromatic luminal cells, high-grade prostatic intraepithelial neoplasia (HGPIN,) and invasive carcinoma compared to the Sh-WT mice. Cx reduced acini size (atrophy) and induced desmoplasia in both WT and TRAMP mice. Notably, I_2_ enhanced the desmoplasia in Sham WT (Sh-WT) mice and led to HGPIN with a cribriform pattern in Sh-TRAMP mice.

Quantitative analyses confirmed that TRAMP tumoral prostates had greater weights and acinar areas compared to those from WT mice (Figure 6B,C). Castration did not alter the prostate wet weight or acinar areas in WT mice; however, it decreased both parameters in TRAMP mice. In both groups, castration reduced the epithelium/acinus ratio (Figure 6D) and increased the stroma/acinus ratio (Figure 6E). As expected, castration also decreased the score of HGPIN lesions in TRAMP mice (Figure 6F) and raised the proportion of mice with higher desmoplasia grading (Figure 6G). Conversely, I_2_ administration increased the percentage and grade of desmoplasia in Sh-TRAMP mice (Figure 6G) but had no effect on most of the other histopathological parameters assessed in this study (Figure 6B–G).

### 2.7. Effects of I_2_ and/or Castration on Immunopositivity for SYP, AR, and PPARG

**Figure 7:**
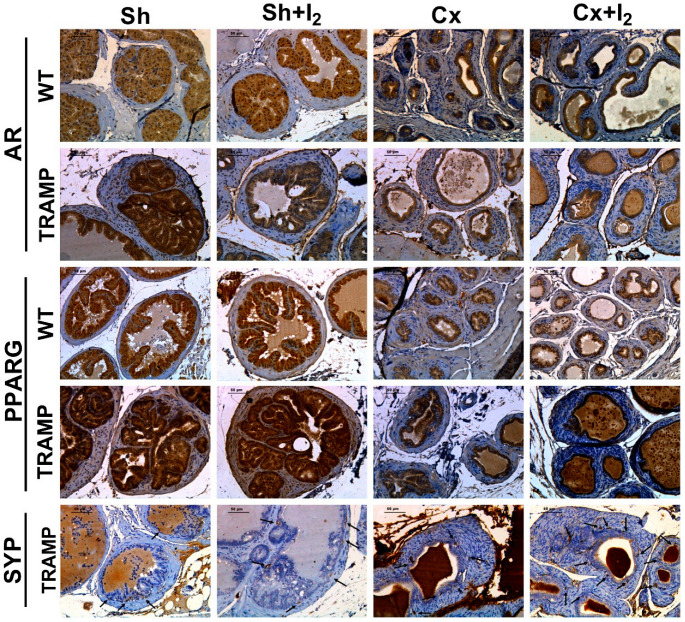
Representative micrographs of immunohistochemistry (IHC) for AR, PPARG, and SYP in the normal (WT) or cancerous (TRAMP) prostates. Positively labeled SYP cells are indicated with black arrows. All images were captured at 20× magnification under light microscopy; scale bar equals 50 µm. Sham (Sh), Castration (Cx).

**Figure 8:**
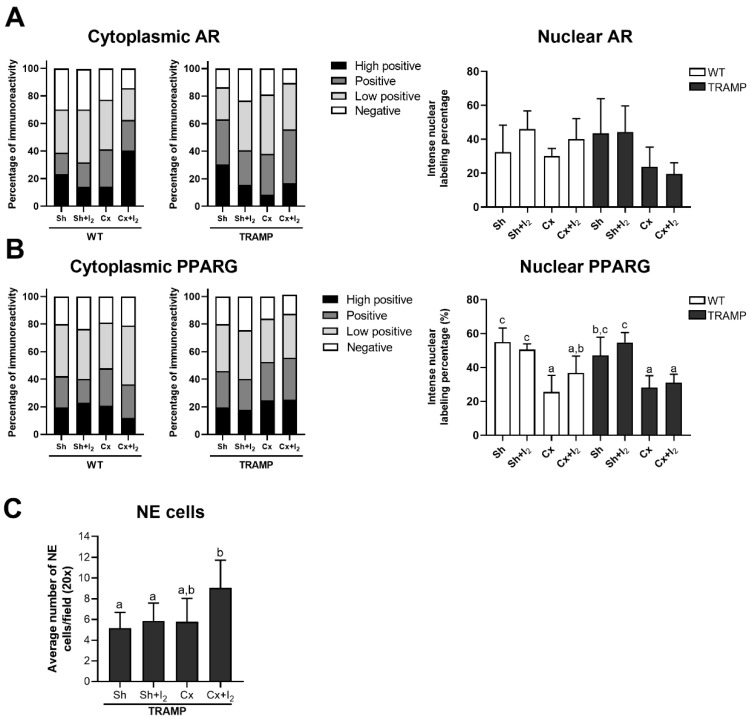
Analysis of IHC for AR, PPARG, and SYP in normal (WT) or cancerous (TRAMP) prostates. (**A**,**B**) Percentage of cytoplasmic immunoreactivity and nuclear labeling per group. (**C**) Quantification of SYP-positive cells/field. Four to five independent fields per mouse were analyzed, *n* = 5–7 mice/group. Data represent SD. A one-way ANOVA followed by a Tukey post hoc test was performed to compare groups. Statistical differences among groups are indicated with different letters (*p* < 0.05); groups that share the same letter are not significantly different.

Finally, we analyzed the effects of iodine and castration on the immunolocalization of PPARG and AR receptors in WT and TRAMP mice, as well as the tumoral prevalence of NE cells (SYP immunodetection) in TRAMP mice. Representative immunohistochemistry (IHC) micrographs of the prostates of WT and TRAMP mice are shown in Figure 7. The quantitative analysis demonstrated that neither treatment significantly altered the cytoplasmic or nuclear AR levels in comparison to their respective sham groups (Figure 8A). In contrast, castration reduced the nuclear immunopositivity for PPARG in both WT and TRAMP mice, while I_2_ had no effect on PPARG in either condition (Figure 8B). Furthermore, the number of SYP-positive cells in TRAMP mice was not affected by castration or I_2_ alone. However, Cx + I_2_ increased SYP immunopositivity compared to the sham group (Figure 8C).

## 3. Discussion

This study showed that I_2_ exhibited in vitro cytotoxic effects and diminished the invasive potential of both androgen-dependent and androgen-independent prostate cancer cell lines. Moreover, I_2_ inhibited the proliferative actions of testosterone and enhanced the cytotoxic effects of antiandrogens, indicating a functional antagonism. Our data showed that I_2_ does not regulate *AR* expression directly; however, it may inhibit its downstream pathway, as the combination of I_2_ and Bic further reduced *KLK3* expression compared to the control group. Additionally, the response observed in androgen-independent prostate cancer cells suggests that the effects of I_2_ may extend beyond androgenic signaling, as its anti-invasive activity has been reported in breast cancer and neuroblastoma models [8,11].

Androgen deprivation therapy is known to inhibit tumor growth, but cancer cells develop long-term resistance mechanisms [2]. Therefore, we analyzed the effects of I_2_ in a long-term androgen deprivation model. As expected, androgen deficiency in LNCaP cells stimulated neurite outgrowth, increased *SYP* and *ENO2* gene expression, and reduced lipid content, indicating the acquisition of an NE-like phenotype and underscoring the role of androgens in lipid metabolism [24,25,26]. In contrast, while I_2_ also promoted neurite projections, it did not increase the expression of NE markers, suggesting the induction of partial or intermediate NE-like differentiation.

The pro-neurite actions of I_2_ were more evident under low-androgen and lipid-sufficient conditions. Consistently, a previous study showed that cytokines, a lipogenic environment, and PPARG activation are essential for neurite-like projection growth in prostate cancer cells [22]. However, in our experiments, the cytotoxic or pro-neurite effects of I_2_ were not PPARG-dependent. Alternative mechanisms, such as reduced catabolism or increased lipid uptake, might explain the elevated lipid content observed in I_2_-treated prostate cancer cells. In contrast, in breast cancer and neuroblastoma, the antitumor effects of I_2_ have been associated with PPARG activation and a lipogenic phenotype [9,11], supporting the idea that I_2_ mechanisms are cell-type specific.

The dual actions of I_2_ on prostate cancer prompted us to explore its effects using the TRAMP model. Previous work from our group showed that chronic I_2_ supplementation (12–24 weeks) did not prevent cancer progression at 18 and 30 weeks of age, possibly due to the aggressive phenotype established in these timeframes [12]. Nonetheless, in this study, we examined whether a four-week co-treatment with I_2_ and castration could modulate tumor growth during the promotion phase.

Our histological results confirmed the well-known androgen dependence of epithelial and stromal compartments in both normal and cancerous prostates [27,28,29]. I_2_ did not affect the morphological parameters (acini size and epithelial/stromal cell ratio), nor did it alter PPARG or AR levels in either sham or castrated mice. Likewise, the epithelial lesion score remained unchanged. However, I_2_ increased tumoral desmoplasia in non-castrated mice and the number of SYP-positive cells in castrated TRAMP prostates. Previous research on neuroblastoma cells (SH-SY5Y) has shown that I_2_ can have neuro-differentiating effects by increasing the number of neurites and inducing the expression of neural NE markers such as NTRK1 [30]. Additionally, increased desmoplasia has been associated with advanced prostate cancer [31], which may partly explain the limited antitumor effects of I_2_ observed in vivo, as a greater presence of NE-like cells within the acini may facilitate tumor persistence under androgen-deprived conditions.

Longitudinal studies are needed to understand how prostatic desmoplasia influences the tumoral microenvironment and metastatic potential. The prostate cancer-associated stroma is known to facilitate cell survival, invasion, and angiogenesis [32]. Our findings show that the antineoplastic effects of I_2_ were insufficient to reduce tumor weight or epithelial lesion scores in castrated mice. This may reflect the inherent aggressiveness of the TRAMP model (due to constitutive SV40 expression) [33], as it contrasts with results obtained with similar I_2_ concentrations in neuroblastoma and breast cancer xenograft models, without generating adverse effects on mouse health or thyroid function [9,34]. Cell cycle arrest, apoptosis, Th1 lymphocyte activation, and CD8+ cytotoxic antitumoral responses are among the antineoplastic mechanisms attributed to I_2_ in vivo in breast cancer [35,36,37]. Although the immunological responses were not fully analyzed in this study, the extent of the desmoplastic reaction and the low presence of infiltrating lymphocytes (Supplementary Figure S3) are limiting factors that could counteract the antitumoral response to I_2_ in the TRAMP model.

We also observed that castration induced prostate inflammation in WT and TRAMP prostates. Interestingly, I_2_ appeared to mitigate this inflammatory response (Supplementary Table S4), which is consistent with its previously reported antioxidant and anti-inflammatory actions in prostatic hyperplasia models [13,38]. These results are consistent with the existing literature, which indicates that late carcinogenesis in TRAMP exhibits an immunosuppressive microenvironment characterized by M2 macrophage recruitment [39], increased macrophage infiltration post-castration [40], and a general classification of prostate cancer as a “cold” tumor due to its low immunogenicity [41]. On the other hand, it has also been demonstrated that I_2_ may influence the epigenetic regulation of inflammatory mediators, particularly by inducing interferon gamma unmethylation [37].

Prostate cancer progression is associated with increased oxidative stress, partly due to reduced nuclear factor erythroid 2-related factor 2 (NRF2)-mediated antioxidant signaling [42]. Notably, I_2_ has been shown to upregulate NRF2 protein levels and nuclear localization [43], suggesting that NRF2 activation could contribute to the anti-inflammatory effect of I_2_ in prostate.

Future studies combining I_2_ with immunotherapy and/or low doses of testosterone (without total deprivation) should be conducted to evaluate possible synergisms and delay prostate cancer progression.

## 4. Materials and Methods

### 4.1. Biological Models

Human prostate cancer cell lines: Androgen-dependent (LNCaP) and androgen-independent (PC-3, DU145, C4-2B) cell lines were provided by ATCC (Manassas, VA, USA). LNCaP cells represent a differentiated cancer model with low invasive potential but capable of undergoing NE differentiation, while PC-3, DU145, and C4-2B cells represent a de-differentiated prostate cancer model with high invasive potential, refractory to androgen deprivation. LNCaP cells were cultured in RPMI, C4-2B cells in DMEM/F-12 (4:1), and PC-3 and DU145 cells in DMEM. RPMI and DMEM were supplemented with 10% FBS and DMEM/F-12 with 10% HI-FBS and T-media. All cells were grown with antibiotics (200 U/mL penicillin and 200 µg/mL streptomycin) at 37 °C in a humidified atmosphere of 5% CO_2_. Cells were used before passage fifteen. Assays are described in the experimental design. Culture media and antibiotics (penicillin/streptomycin) were provided by Gibco (Grand Island, NY, USA), and FBS types were provided by Biowest (Bradenton, FL, USA).

TRAMP model: Male C57BL/6 WT and TRAMP (SV40+/−) mice were genotyped using PCR, as previously reported [12]; the primers employed are listed in Supplementary Table S1. SV40 large T antigen is an inhibitory oncoprotein of the transformation-related protein 53 (Trp53/p53) and retinoblastoma transcriptional corepressor 1 (Rb1) proteins [44]. Additionally, through the inactivation of protein phosphatase 2A (PP2A) mediated by SV40 short T antigen, it induces prostate carcinogenesis [45].

### 4.2. Androgen and PPARG Inhibition

Androgen signaling was blocked with specific antagonists: Bic (Cayman Chemical 14250; Ann Harbor, MI, USA) and Enz (Selleckchem S1250; Houston, TX, USA). Androgen deprivation was achieved by supplementing RPMI media with 10% charcoal-stripped FBS (CS-FBS, Biowest S181F; Bradenton, FL, USA). Testosterone propionate (Sigma-Aldrich T1875; St. Lous, MO, USA) was used as a positive control to stimulate the AR pathway. PPARG activation was inhibited with the antagonist GW9662 (Sigma-Aldrich 6191). For the in vivo study, androgen deficiency was induced in 18-week-old WT and TRAMP mice by surgical castration under anesthesia with a mixture of ketamine/xylazine (75 and 0.6 mg/kg of body weight). Control mice were subjected to sham surgeries consisting of scrotal incisions and wound closure without gonad removal. Every experimental procedure performed on the mice was reviewed and approved by the Research Ethics Committee at the Instituto de Neurobiología, UNAM.

### 4.3. Research Design

#### 4.3.1. Effects of I_2_ on Cell Viability and Invasive Capacity

LNCaP, C4-2B, PC-3, and DU145 cells were seeded at a 10,000–20,000 cm^2^ density in 12-well culture plates and cultured with I_2_ (100 to 800 µM) for 96 h to analyze cell viability. On the other hand, LNCaP, PC-3, and DU145 cells were pre-treated with 200 or 400 µM of I_2_ for 48 h, and the invasive capacity was evaluated by Transwell chambers in 6 h assays.

#### 4.3.2. Effects of I_2_ on the Androgen Antagonist Response

Cell viability was evaluated in cultures (10% FBS) of LNCaP and DU145 cells (negative control) treated with different concentrations of Bic (0.5 to 10 µM) or Enz (0.01 to 10 µM) for 96 h. We analyzed the individual and combined effects of I_2_ and/or Bic, and I_2_ and/or Enz at their respective IC_50_ concentrations on the viability of LNCaP cells at 96 h. I_2_ and/or Bic effects were also evaluated under stimulation with 10 nM testosterone for 96 h. The effects of I_2_ and/or Bic on AR, as well as on AR target genes (*KLK3*, *NKX3-1*), were evaluated by real-time PCR at 48 h of treatment. DMSO and absolute ethanol were used as vehicles for the AR antagonists and testosterone propionate, respectively.

#### 4.3.3. The Effects of I_2_ on the Acquisition of a NE-like Phenotype Induced by Androgen Deprivation in LNCaP Cells

Cells were cultured for 3, 10, and 15 days in either standard (10% FBS) or androgen-deprived conditions (10% CS-FBS) with or without 200 µM I_2_. The NE phenotype was identified by the growth of neurite-like projections and the expression of NE markers such as *SYP* and *ENO2*. The length of neurite-like projections was assessed at different times (3, 10, and 15 days) using phase-contrast microscopy (OLYMPUS IX50; Center Valley, PA, USA) and ImageJ software (NeuronJ plugin, version 1.4.3). The length of these projections was expressed as a fold change regarding the basal length of the cell projections. Real-time PCR was used to analyze NE gene expression. In addition, neutral lipid accumulation (Oil Red O staining) was measured on the 10th day of treatments. DU145 cells served as a negative control for androgen signaling and were treated with 400 µM I_2_ for neutral lipid staining.

#### 4.3.4. Influence of Androgenic Status and Contribution of PPARG to I_2_ Effects

LNCaP cells were cultured with 0.1, 1.0, and 10 µM of the PPARG antagonist (GW9662) under standard conditions (10% FBS), and viability was evaluated at 96 h. Then, LNCaP cell viability was assessed after they were co-cultured for 96 h with 200 µM of I_2_ and 0.1 or 1 µM of PPARG antagonist (GW9662) under standard or androgen-deprived conditions (10% CS-FBS). In both conditions, the effects of I_2_ on the expression of PPARG target genes, such as *FASN* and *SREBF1*, were evaluated by real-time PCR. The contribution of PPARG to the growth of NE-like projections was examined in LNCaP cells cultured under androgen deprivation and co-treated with 200 µM I_2_ and 1 µM GW9662 for 10 days. Individual treatments with I_2_ in standard or androgen-deprived conditions were used as controls.

#### 4.3.5. The Effects of I_2_ and Androgen Deficiency on the Prostatic Regulation of AR and PPARG and Cancer NE Progression in TRAMP Mice

I_2_ was added to the drinking water (0.025% final concentration) and administered for four weeks to 18-week-old mice. The following four groups for WT or TRAMP mice were formed: Sh (sham), Sh + I_2_, Cx (castrated), and Cx + I_2_. Four weeks later, the 22-week-old mice were euthanized, and both normal and cancerous prostates were processed for histopathological (hematoxylin–eosin and Masson’s trichrome) or immunohistochemical analysis of SYP, AR, and PPARG.

### 4.4. Analytical Methods

#### 4.4.1. Cell Viability

Cells were seeded in 12-well culture plates. The media were removed 24 h later, and fresh medium with or without treatments was added. Viable cells were counted using the trypan blue exclusion assay. The data were transformed into percentages of change compared to the initial cell count (time course) or normalized against the non-treated control (96 h).

#### 4.4.2. Intracellular Lipid Content

Cells were fixed with 10% formalin for 30 min and stained with a 0.018% *w*/*v* isopropanol Oil Red O solution (Sigma-Aldrich O0625). Stained cells were then photographed under light microscopy, and red fluorescence was quantified by ImageJ as an indicator of lipid abundance, as previously reported [46]. Four independent fields were analyzed from three independent experiments.

#### 4.4.3. Gene Expression

We analyzed gene expression with real-time PCR. Total RNA was extracted using TRIzol reagent (Thermo Fisher Scientific 15596026; Waltham, MA, USA), and its integrity was verified by analyzing the spectrophotometry absorbance 260/280 and 260/230 ratios; both ratios were ≥1.8. RNA (2 µg/sample) was retrotranscribed with oligo dT primer and the M-MLV kit (Invitrogen 28025013; Waltham, MA, USA). PCR was performed using SYBR Green/ROX Master Mix (Invitrogen, K0223). Thermocycler conditions were as follows: 40 cycles at 95 °C for 15 s, 55–62 °C for 30 s, and 72 °C for 30 s. Primer specificity was confirmed by a melting curve and amplicon size was determined by electrophoresis. No products were amplified in the absence of cDNA. Gene expression was normalized using the expression of beta-actin (*ACTB*) or glyceraldehyde-3-phosphate dehydrogenase (*GAPDH*) as an internal loading control using a standard curve or the 2^−ΔΔCT^ method, respectively. Characteristics of the primers are depicted in Supplementary Table S2.

#### 4.4.4. Invasion Assays

LNCaP, DU145, and PC-3 cells were previously treated with I_2_ for 48 h and 2.5 × 10^4^ cells were seeded in Matrigel^®^ Invasion Chambers (Corning 354480; Corning, NY, USA). Five percent FBS-supplemented medium was used as a chemoattractant in the lower chambers. Six hours later, invading cells were fixed with methanol and stained with 0.5% toluidine blue and 0.5% borax solution and counted in seven random fields under light microscopy.

#### 4.4.5. Histopathology Analysis

Prostates were fixed in formalin solution and later subjected to dehydration and paraffin-embedded sectioning. Sections of 3 µM in thickness were deparaffinated, rehydrated, and stained with hematoxylin–eosin. We assessed the epithelial and stromal ratios with respect to acini size in all mice and estimated the severity of epithelial lesions in TRAMP (distribution-adjusted lesion) as previously reported [47]. We classified the extent of proliferative epithelia and prevalence of lesions (focal, multifocal, or diffuse) as hyperplasia (score 1–3), adenoma (score 4–5), or adenocarcinoma (score 6).

In addition, tumoral slides were subjected to Masson’s trichrome staining to assess the degree of desmoplasia, considering stromal growth and the loss of basal membrane integrity. The prevalence of desmoplasia in mice was calculated using the sum of two criteria: the number of membrane disruption foci per field and the stroma/acinus ratio, which measures the growth of stroma. Desmoplasia was classified accordingly as null (0), low (1), mid (2), and high (≥3).

#### 4.4.6. Immunohistochemistry

The antigen was retrieved with a heat-pressure cooker in sodium citrate buffer (10 mM, pH 6). Endogenous peroxidases were blocked with 3% H_2_O_2_. Unspecific antibody binding was reduced through 1 h incubation with 3% BSA and 3% T-PBS (X-100 Triton phosphate-buffered saline). Slides were incubated overnight with the indicated primary antibodies diluted in T-PBS. After 3 × T-PBS rinses, slides were incubated with their corresponding secondary antibodies for 2 h. Then, avidin peroxidase was coupled to biotinylated secondary antibodies for 1 h incubation in the dark with ABC reagents (Vectastains PK-6101 & PK-6102; Vector laboratories, Newark, CA, USA), followed by 3 × PBS rinses and incubation with DAB substrate (Vector laboratories SK-4100). Slides were counterstained with hematoxylin before being dehydrated and mounted with Entellan^TM^. Micrographs were captured with a Leica DM2500 microscope and a DFC420 camera. Antibody characteristics and dilutions employed for IHC are detailed in Supplementary Table S3.

#### 4.4.7. IHC Immunolabeling Quantification

Nuclear and cytoplasmatic AR and PPARG immunolabeling were assessed with the ImageJ IHC Profiler plugin, which performs a color deconvolution to separate the hematoxylin signal (nucleus) from the DAB signal [48]. Micrographs were pre-processed to remove non-specific backgrounds. The distribution of cytoplasmic labeling was evaluated and classified as negative, low positive, positive, and high positive. For nuclear IHC quantification, we established a Huang densitometric threshold at (0, 60) in the IHC Profiler nuclear-stained image module to quantify the percentage of intense labeling. NE-like cells were identified by counting SYP-positive cells within acini. In all cases, four to five independent fields per mouse were captured and analyzed at 20× magnification.

### 4.5. Statistical Analysis

Invasive capacity was analyzed using a Student’s *t*-test. Cell viability, gene expression, the length of neurite-like projections, histological parameters, and IHC were analyzed with one-way or two-way ANOVA followed by a Tukey post hoc test. Sample sizes are indicated in figure legends. Different letters or asterisks indicate significant differences between groups (*p* < 0.05). All statistical analyses were performed using GraphPad Prism v. 6.01 (GraphPad Software Inc.; San Francisco, CA, USA). 

## 5. Conclusions

This study demonstrated that I_2_ exerted cytotoxic, anti-invasive, pro-neurite, and lipogenic effects in prostate cancer cells, as well as induced desmoplastic effects in prostate tumors. The anti-tumor and pro-tumor effects of I_2_ were independent of androgens and PPARG. Additional studies should be conducted to understand the phenotypic and metabolic responses to I_2_ in prostate carcinogenesis. The halogenation of biomolecules, redox balance, and epigenetic regulation are examples of potential direct mechanisms of I_2_ that warrant further study in cancer and beyond.

## Data Availability

Dataset available on request from the authors.

## Supplementary Materials

The supporting information can be downloaded at: https://www.mdpi.com/article/10.3390/ijms26167800/s1.

## Abbreviations

The following abbreviations are used in this manuscript:

ACTB	Beta-actin
AR	Androgen Receptor
ENO2	Enolase 2
FASN	Fatty Acid Synthase
GAPDH	Glyceraldehyde-3-Phosphate Dehydrogenase
HGPIN	High-Grade Prostatic Intraepithelial Neoplasia
KLK3	Kallikrein-related peptidase 3
NE	Neuroendocrine
NKX3-1	NK3 homeobox 1
PCNA	Proliferating Cell Nuclear Antigen
PPARG	Peroxisome Proliferator-Activated Receptor Gamma
SREBF1	Sterol Regulatory Element Binding Transcription
SYP	Synaptophysin
TRAMP	Transgenic Adenocarcinoma of the Mouse Prostate
