# Supplementary material for: Molecular Iodine Induces Anti- and Pro-Neoplastic Effects in Prostate Cancer Models
Source: Int J Mol Sci. 2025 Aug 13;26(16):7800. doi: 10.3390/ijms26167800 (PMC12386490; doi:10.3390/ijms26167800)
Supplement: Supplementary file 1 [file ijms-26-07800-s001.zip › ijms-3750663-supplementary.pdf]

Supplementary figures

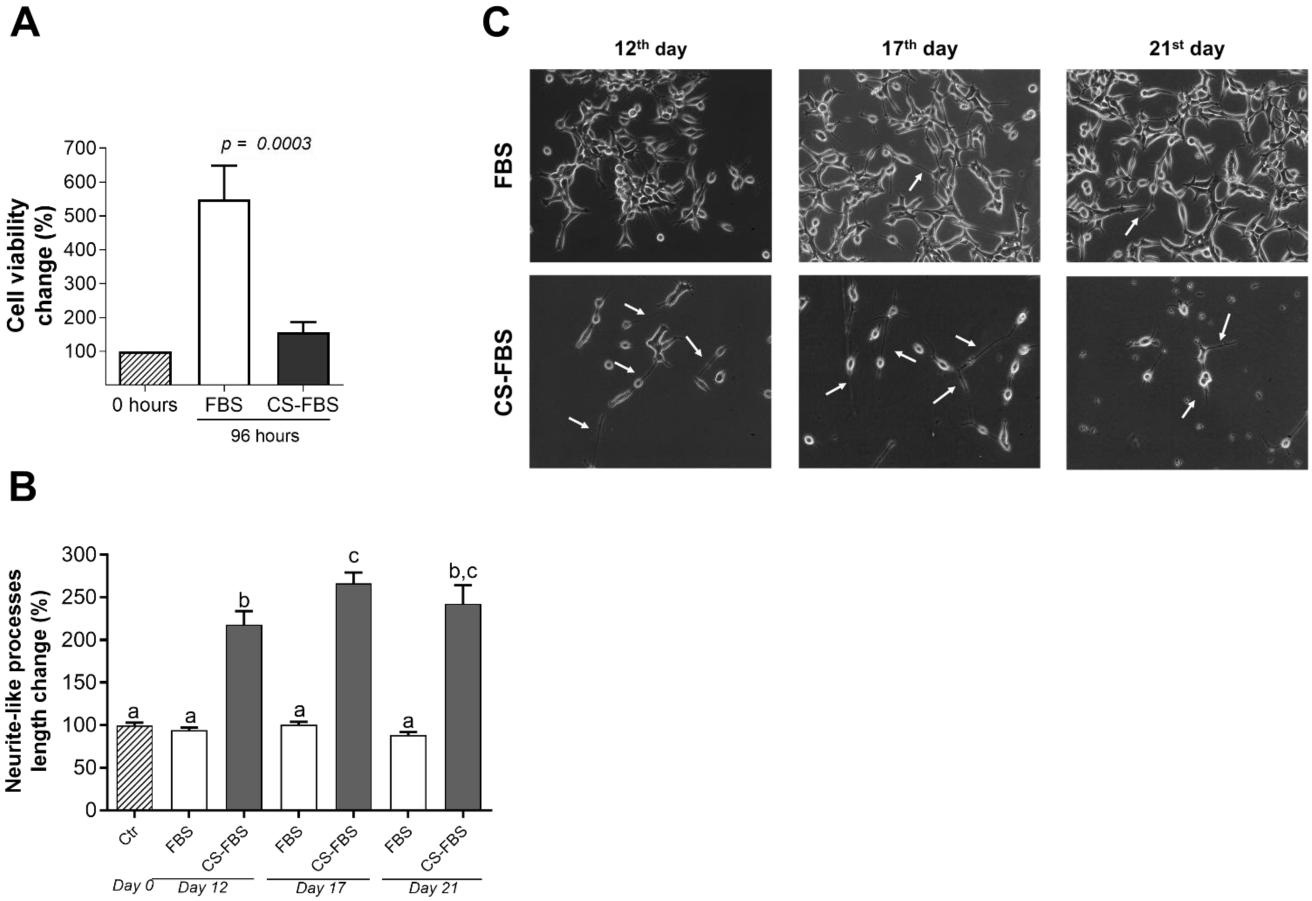

Supplementary Figure S1. A Effects of androgen depletion (charcoal-stripped fetal bovine serum, CS-FBS) on cell viability. Data was normalized to time zero in 96 h cultures. **B** Effects of CS-FBS on neurite outgrowth. The time course for neurite-like process length was normalized to time zero in cells cultured under androgen deprivation. **C** Representative micrographs of LNCaP cells. Neurite-like projections are shown (arrows). Images were captured at 200x magnification using a bright-field microscope. Cell proliferation was analyzed with an unpaired t-test, n = 4 independent experiments in duplicate. Neurite-like processes were analyzed with a one-way ANOVA followed by a Tukey post hoc test (n = 3 independent experiments). Different letters indicate statistical differences among groups. ($p < 0.05$).

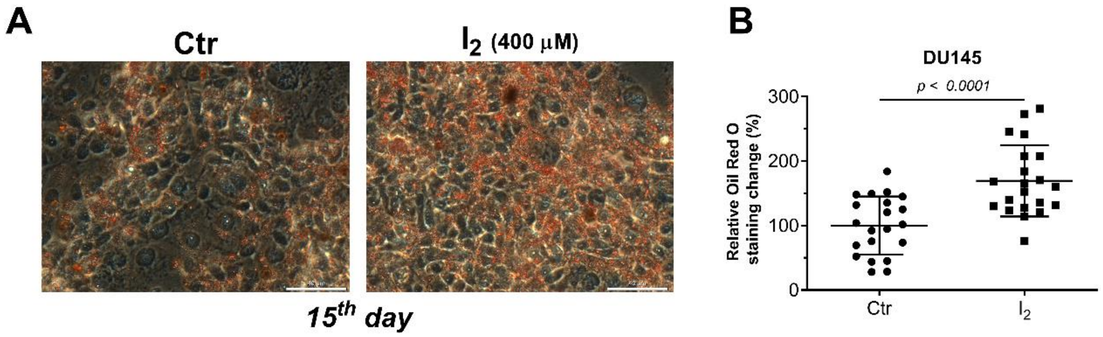

Supplementary Figure S2. **A** Effects of I_2 on neutral lipid content in DU145 cells. a Representative micrograph from DU145 cells were cultured in the presence or absence of I_2 (400 μM) for 15 days. Images were captured at 20x magnification using a bright-field microscope. Scale bar; 40 μm. **B** Quantification of lipids was performed by densitometry in seven fields. Data represent mean ± SD and were analyzed with a t-test. n = 3 independent experiments.

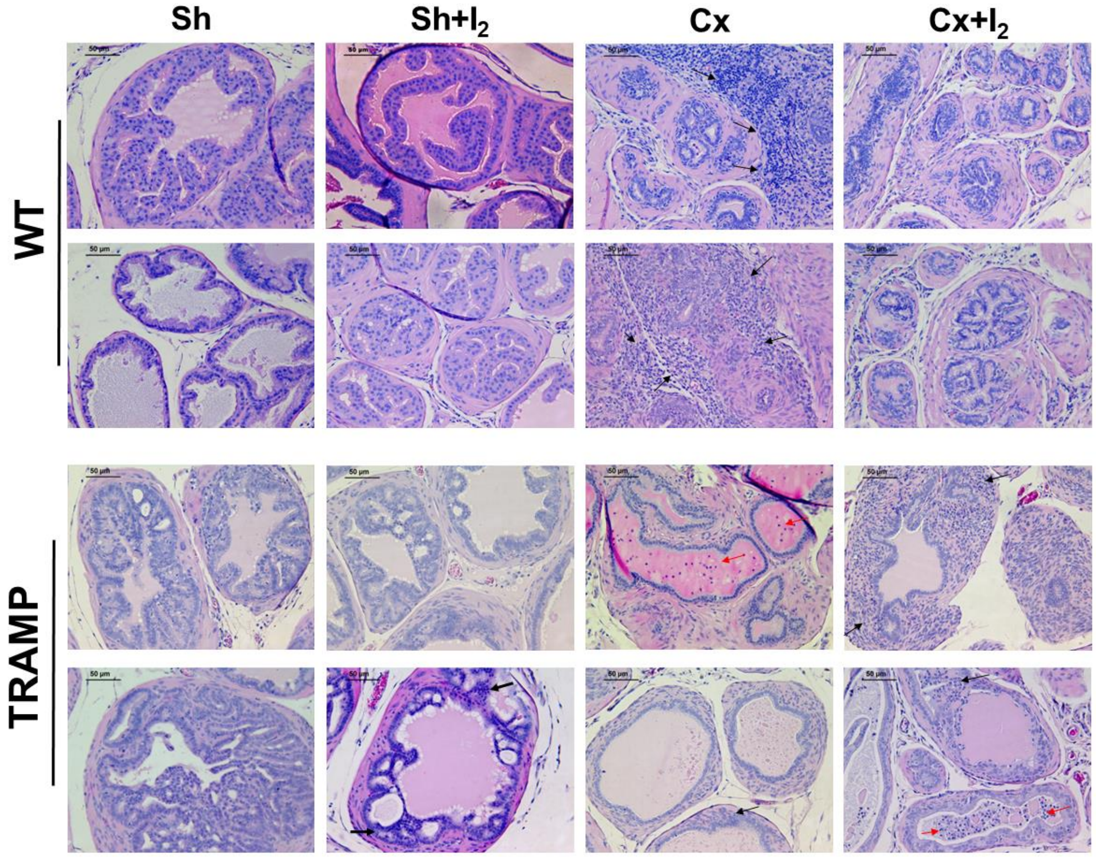

Supplementary Figure S3. Effect of I_2 and/or castration on prostate inflammation. Representative micrographs of normal (WT) and cancerous (TRAMP) mice. Black arrows highlight regions with desmoplasia. Red arrows highlight regions with infiltrating lymphocytes. All images were captured at 20x magnification under light microscopy. The scale bar equals 50 μm.

Supplementary tables

Supplementary Table S1. Primers employed for genotyping of TRAMP mice.

Target	Primer sequences	Product size (pb)
SV40	**S**: CAG AGC AGA ATT GTG GAG TGG<br>**AS**: GGA CAA ACC ACA ACT AGA ACT AGA ATG CAG TG	500
TRCD	**S**: CAA ATG TTG CTT GTC TGG TG<br>**AS**: GTC AGT CGA GTG CAC AGT TT	200

Supplementary Table S2. Primers employed for real-time PCR.

Classification	Gene	RefSeq ID	Primer sequences	Product size (pb)
**Nuclear Receptors**	***AR***	NM_000044.4<br>NM_001348063.1<br>NM_001348061.1<br>NM_001011645.3	**S**: GAC CTT ACG GGG ACA TGC G<br>**AS**: TTC CCT TCA GCG GCT CTT TT	175
	PPARG	NM_138712.5<br>NM_001374266.1<br>NM_138711.6	**S**: GAC CAC TCC CAC TCC TTT GA<br>**AS**: TTCGA CAT TCA ATT GCC ATG AG	257
**PPARG target genes**	***FASN***	NM_004104.5	**S**: ATG CTG AAC GAC ATC GCG G<br>**AS**: GAA TCT CGG AAG CGG TCC AG	200
	SREBF1	NM_001005291.3<br>NM_001321096.3	**S**: AGA TCG CGG AGC CAT GGA TTG<br>**AS**: CTG CCT GGG GAG CTG GTA TC	153
**AR target genes**	***NKX3.1***	NM_006167.4<br>NM_001256339.1	**S**: AAG AGA ACG CCC TCA TGC TC<br>**AS**: TTC TAT TTG GGC CAC CCT GT	146
	KLK3 (PSA)	NM_001648.2<br>NM_001030048.1	**S**: AAG CTG GAG GCA CAA CGC ACC<br>**AS**: CCT CCT TGG CTC ACA GCC TTC TC	111
**NE markers**	***SYP***	NM_003179.22	**S**: CTG TGA CCT CGG GAC TCA AC<br>**AS**: GTA GCC TGC ATC GCC GTA	179
	ENO2 (NSE)	NM_00175.3	**S**: AGC TGA GGG ATG GAG ACA AAC<br>**AS**: CAT CCA ACT CCA GCA TCA GGT	153
**Housekeeping genes**	***GAPDH***	NM_002046.7<br>NM_001256799.3<br>NM_001289745.3<br>NM_001289746.2<br>NM_001357943.2	**S**: GAC AAC TTT GGC ATC GTG GA<br>**AS**: ATG CAG GGA TGA TGT TCT GG	133
	ACTB	NM_001101.4	**S**: GTC CAG TAT GCC TCT GGT CGT AC<br>**AS**: CAC GCT CGG TCA GGA TCT TCA TG	169

Supplementary Table S3. Antibodies employed for immunohistochemistry.

Primary antibodies				
**Target**	**Specificity**	**Supplier**	**Cat. number**	**Dilution employed**
**SYP**	Mouse monoclonal	Santa Cruz Biotechnology	sc-17750	1:500
**AR**	Rabbit polyclonal	Santa Cruz Biotechnology	sc-816	1:500
**PPARG**	Rabbit polyclonal	Sigma-Aldrich	SAB5700625	1:600

Secondary antibodies			
**Target species & labeling**	**Supplier**	**Cat. number**	**Dilution employed**
Anti-rabbit Biotinylated	Vector Laboratories	PK-6101	1:1000
Anti-mouse Biotinylated	Vector Laboratories	PK-6102	1:1000

Supplementary Table S4. Percentage of mice exhibiting acini inflammation.

**Prevalence of inflammation**								
	**WT**				**TRAMP**			
	**Sh**	**Sh+I_2**	**Cx**	**Cx+I_2**	**Sh**	**Sh+I_2**	**Cx**	**Cx+I_2**
**Cases/total**	1/7	0/5	4/4	2/4	1/6	3/7	5/6	1/5
**Percentage**	14.3%	0%	100%	50%	16.7%	42.8%	83.3%	20%

Four independent fields per mice were analyzed. **Sh**, sham; **Cx**, castration.
